# Short axis versus axial Cine SSFP MR imaging for assessment of right and left ventricular function: intrapatient correlation with phase-contrast flow measurements

**DOI:** 10.1186/1532-429X-13-S1-P40

**Published:** 2011-02-02

**Authors:** Susan H James, Rachel Wald, Bernd J Wintersperger, Laura Jimenez-Juan, Djeven Deva, Andrew Crean, Elsie Nguyen, Narinder Paul, Sebastian Ley

**Affiliations:** 1 Department of Medical Imaging;Toronto General Hospital, Toronto, ON, Canada; 2 Department of Medicine and Pediatrics, Division of Cardiology; Toronto General Hospital, Toronto, ON, Canada

## Objective

To compare RV and LV volumetric measurements based on short-axis-oblique (SAO) and axial orientation Cine-SSFP imaging in comparison to respective PC flow measurements in the main-pulmonary-artery (MPA) and ascending-aorta (Aorta).

## Background

MRI is deemed standard of reference for assessment of ventricular volumes and function. However, it remains unclear if the RV can also be adequately assessed in SAO orientation as used for assessment of LV function or requires dedicated axial-Cine imaging. Additional axial-Cine acquisition adds 10-15 min of scan time and thus has a substantial impact on clinical workflow and patient comfort.

## Methods

Retrospective database analysis for patients undergoing CMR for assessment of possible cardiac shunts with MR exclusion of shunts identified 27 subjects (12male/15female) eligible for further data analysis. Based on echo no valvular disease was evident. Patients underwent CMR on a 1.5T system (Magnetom Avanto, SiemensHealthcare) with retrospective gated Cine-SSFP (25 frames/RR) in axial and SAO slice prescription. Spatial resolution for SAO and axial Cine-SSFP was 1.25x1.25x8mm^3^, no slice gap. Semi-automated analysis of RV and LV volumes were performed on axial and SAO-Cine data by a single observer as well as PC flow analysis of MPA and Aorta-flow as a reference measure of RV and LV output (syngo ARGUS, SiemensHealthcare).

## Results

RV-axial (81±18ml) and RV-SAO (76±17ml) stroke volumes showed no significant difference (p=0.1, t-test). There was a high linear correlation between MPA-PC-flow measurements and RV-stroke volume determined in SAO (r=0.80) and axial (r=0.85) orientations. Bland-Altman-Analysis revealed an offset of 1.6 ml (limits: 26 to -23ml) for RV-axial versus MPA-PC-flow and -3.9 ml (limits: 23 to -31ml) for RV-SAO versus MPA-PC-flow.

Stroke volumes of the LV assessed in SAO (81±18ml) and in axial (82±22ml) orientation showed no significant difference (p=0.6). There was a high linear correlation between the LV-SV in SAO (r=0.89) and axial (r=0.88) orientations compared to Aorta-PC-flow measurements. Bland-Altman-Analysis revealed an offset of 8 ml (limits: 24 to -8ml) for LV-SAO versus Aorta-PC-flow and 9 ml (limits: 30 to -12ml) for LV-axial versus Aorta-PC-flow.

There was no significant difference (p=0.9) between the SAO-LV-SV and the axial RV-SV (linear correlation r=0.88). There was no significant difference (p=0.1) between the SAO-LV-SV versus SAO-RV-SV (linear correlation r=0.75).

## Conclusion

The results of this initial study demonstrate no significant impact of the slice acquisition orientation for determination of right and left ventricular stroke volumes. CMR workflow could therefore be streamlined for most cases only acquiring SAO cine images for ventricular volumetrics.

